# STATE-LEVEL DESCRIPTION OF MEDICAID POLICY AND ACCESS TO LICENSED ASSISTED LIVING CARE FOR LOW-INCOME ADULTS

**DOI:** 10.1093/geroni/igad104.3602

**Published:** 2023-12-21

**Authors:** Rachel Gunderson, Lindsey Smith, Portia Cornell, Gauri Gadkari, Kali Thomas

**Affiliations:** Brown University, Providence, Rhode Island, United States; Oregon Health & Science University, Portland, Oregon, United States; Providence VA Medical Center, Providence, Rhode Island, United States; Brown University, Providence, Rhode Island, United States; Johns Hopkins University, Baltimore, Maryland, United States

## Abstract

A growing proportion of Medicaid funds are allocated to care in assisted living (AL) settings. However, a state must have a state plan amendment or waiver to utilize these funds. States issue levels of licensure for AL communities, which govern the care and oversight that can be provided and may determine whether ALs can accept Medicaid funding. However, until now, Medicaid coverage for AL has been analyzed at the state level and is not well-documented for license types. Using Medicaid state plans and waiver applications, we describe the types of Medicaid funding mechanisms that states use to finance care in AL and identify the demographics and health characteristics of residents in Medicaid-eligible ALs. We find that five states do not utilize Medicaid to finance care in AL. Among the 45 states and DC with Medicaid-financed AL, 12 states limit Medicaid funding to specific licenses, whereas 34 states provide access to Medicaid-funded care for all AL license types. Among identified ALs, 86% are licensed to accept Medicaid funds. Although the modal funding mechanism is through 1915c waivers, the largest percentage (40%) of ALs licensed to accept Medicaid funding are in states that utilize multiple mechanisms. In states that fund AL through state plan amendments, 88% of Medicare beneficiaries reside in covered ALs; conversely, in states that use 1115 demonstration waivers, 99% of Medicare beneficiaries reside in covered ALs. This work will benefit future research and policymaking aiming to use Medicaid funding to increase access to appropriate, high-quality care in licensed ALs.

